# *S*-Locus Genotyping in Japanese Plum by High Throughput Sequencing Using a Synthetic *S*-Loci Reference Sequence

**DOI:** 10.3390/ijms24043932

**Published:** 2023-02-15

**Authors:** Afif Hedhly, María Engracia Guerra, Jerome Grimplet, Javier Rodrigo

**Affiliations:** 1 Departamento de Ciencia Vegetal, Centro de Investigación y Tecnología Agroalimentaria de Aragón (CITA), Avda Montañana 930, 50059 Zaragoza, Spain; 2 Área de Fruticultura Mediterránea, CICYTEX-Centro de Investigación ‘Finca La Orden-Valdesequera’, A-V, KM 372, Guadajira, 06187 Badajoz, Spain; 3 Instituto Agroalimentario de Aragón-IA2, CITA-Universidad de Zaragoza, 50013 Zaragoza, Spain

**Keywords:** self-incompatibility, *S*-allele, *S*-genotyping-by-sequencing, *Prunus salicina*, Japanese plum

## Abstract

Self-incompatibility in *Prunus* species is governed by a single locus consisting of two highly multi-allelic and tightly linked genes, one coding for an F-box protein—i.e., SFB in *Prunus*- controlling the pollen specificity and one coding for an *S-RNase* gene controlling the pistil specificity. Genotyping the allelic combination in a fruit tree species is an essential procedure both for cross-based breeding and for establishing pollination requirements. Gel-based PCR techniques using primer pairs designed from conserved regions and spanning polymorphic intronic regions are traditionally used for this task. However, with the great advance of massive sequencing techniques and the lowering of sequencing costs, new genotyping-by-sequencing procedures are emerging. The alignment of resequenced individuals to reference genomes, commonly used for polymorphism detection, yields little or no coverage in the *S*-locus region due to high polymorphism between different alleles within the same species, and cannot be used for this purpose. Using the available sequences of Japanese plum *S*-loci concatenated in a rosary-like structure as synthetic reference sequence, we describe a procedure to accurately genotype resequenced individuals that allowed the analysis of the *S*-genotype in 88 Japanese plum cultivars, 74 of them are reported for the first time. In addition to unraveling two new *S*-alleles from published reference genomes, we identified at least two *S*-alleles in 74 cultivars. According to their *S*-allele composition, they were assigned to 22 incompatibility groups, including nine new incompatibility groups reported here for the first time (XXVII-XXXV).

## 1. Introduction

In flowering plants, Gametophytic Self-Incompatibility (GSI) is the most widespread and ancient post-pollination mechanism of non-self and self-recognition that inhibits the growth of self-pollen tubes in the style, preventing self-fertilization [1,2]. Self-incompatibility allows maintaining genetic diversity and avoiding the potentially negative effects of inbreeding [3]. It is controlled by a single genetic locus subjected to strong negative frequency selection, i.e., rare alleles have a mating advantage, [4] that would lead to an increase in the number and diversity of *S*-alleles [5]. The *S*-locus consists of two tightly linked genes that determine both pollen and pistil specificity [6]. In *Prunus* sp., as in other species of the Rosaceae, pollen allele specificity is determined by an F-box protein (SFB) that is expressed in the pollen grain [7], and pistil allele specificity is determined by a ribonuclease (S-RNase) that is expressed in styles [8,9], and inhibits the growth of self-pollen tubes [10]. The genotyping of *S*-locus in both natural and domesticated plant species has confirmed the high allelic diversity and sequence polymorphism [11,12].

In fruit trees, *S*-allele genotyping is of great practical and scientific importance, since it allows the adequate design of crosses in breeding programs and the correct choice of pollinators for self-incompatible cultivars in new orchards [13]. In Japanese plum (*Prunus salicina* and hybrids of *P. salicina* with other *Prunus species*), most cultivars are self-incompatible and require cross-pollination to set fruit. Therefore, knowledge of the *S*-genotype of each cultivar is essential for the design of new orchards with an adequate proportion of inter-compatible cultivars and to solve problems of low fruit set related to lack of pollination [14,15]. It also allows the characterization of allelic series in natural populations to advance our understanding of gene flow and the evolution of mating barriers in plants, and their ecological and evolutionary implications [16,17].

In fruit tree species, self-incompatibility and inter-incompatibility relationships between cultivars were initially determined on the basis of controlled crosses followed by characterization of fruit set under orchard conditions [18]. Later, incompatibility was determined more precisely by microscopic observation of pollen tube growth through the style in self- and cross-pollinated flowers [19], first in the field and later in semi-in vivo cut flowers in the laboratory (e.g., [20,21,22]). These procedures allowed the characterization of cultivar self-(in)compatibility and the establishment of incompatibility groups by using an arbitrary nomenclature system of *S*-alleles. Towards the end of the 20th century, the identification of proteins (S-RNases and SFBs) associated with *S*-alleles allowed the identification of *S*-genotypes by biochemical methods [23,24]. Likewise, the cloning and sequencing of the genes encoded by the *S*-locus allowed obtaining the first *S*-allele sequences upon which different PCR techniques evolved (reviewed in [25,26]). In *Prunus* and other genera belonging to the Rosaceae, degenerate primers designed from conserved exonic regions within the *S-RNase* and *SFB* genes and spanning polymorphic intronic regions allowed trans-specific *S*-genotyping using only a few primer pairs [27]. In the absence of intronic length polymorphism, other PCR-based techniques were developed, such as the single strand conformation polymorphism (SSCP) technique [28]. Nowadays, the tremendous advances in whole genome sequencing are opening new avenues for less time- and resource-consuming *S*-genotyping procedures. However, reference genomes, which are currently available for many economically important fruit tree species, cannot be used to accurately align and resequence the *S*-genotype of individuals due to the extreme polymorphism that characterizes the *S*-locus. To circumvent this pitfall, several approaches have recently emerged, including methods based on bar-coded amplicons [29,30,31], separate alignment on several reference genomes of the same species [32], and *de novo* assemblies of either the whole genome, followed by BLAST and phylogenetic analyses [31], or the *S*-allele itself [33,34]. In apple (*Malus domestica*), de Franceschi et al. managed to *S*-genotype 63 resequenced cultivars using 32 apple *S-RNase* sequences concatenated in a string-like structure as reference sequence [33].

In this work, building on the previously described procedure used in *M. domestica* [33], we present an improved methodology to use information from the entire range of known *S*-loci (genomic regions including *S-RNases* and *SFBs*), rather than just one of the two genes that form the locus, for successful *S*-genotyping 88 individual shotgun sequences of Japanese plum cultivars. To circumvent the problem of unspecific high depth of coverage in intronic regions, which can lead to misidentification of alleles, we developed a simple but quantitative three-step validation that relies not only on the depth but also on the breadth of coverage. This methodology allowed us to accurately ascertain the *S*-genotype of most cultivars, enabling accurate *S*-genotyping of any individual (i.e., the four alleles) as long as it has an already known *S*-allele, and to identify individuals with supernumerary *S*-alleles, or with likely novel and unknown alleles.

## 2. Results and Discussion

### 2.1. An S-Locus Based Synthetic Sequence

Since the first two Japanese plum *S-RNase* alleles were cloned in 1999 from the Japanese cultivar ‘Sordum’ [35], a further 53 *S-RNases* and 36 *SFBs* sequences—corresponding to 39 different loci—have been published or submitted directly to the NCBI database (Table 1 and references therein) representing Chinese, Japanese, North American, Israeli, Hungarian, and Tunisian germplasms. As this is a locus involved in mate recognition, a high degree of diversification and high number of alleles was suggested and confirmed in both wild and domesticated plant species [5,36,37]. Further genotyping of germplasm from China—where the species is believed to have originated—will undoubtedly reveal more polymorphism. To confirm the identity of all *S*-alleles and detect possible duplicates, i.e., redundant or inconsistent-, we performed phylogenetic and pairwise similarity analyses at the nucleotide and amino acid levels for all alleles (data not shown). We detected some redundancies, such as that the *S*_1_, *S*_3_, *S*_4_, *S*_5_, and *S*_6_ alleles were actually duplicates of the *S_a_*, *S_k_*, *S_c_*, *S_e,_* and *S_f_* alleles, respectively (Table 1). There was one case of two different *S*-alleles receiving the same name (*S*_23_-RNase), and both sequences were conserved. Despite the high similarity between *S*_10_ and *S*_32_ (99.3% identity at the nucleotide level), we could not fully determine whether they are the same allele. There was only 96.0% identity at the amino acid level, differing at the 3 amino acids immediately downstream of the conserved RC4 region. In addition, both arrangements are shared with other *S*-alleles. Both alleles were also conserved to build the synthetic sequence.

**Figure 1:**
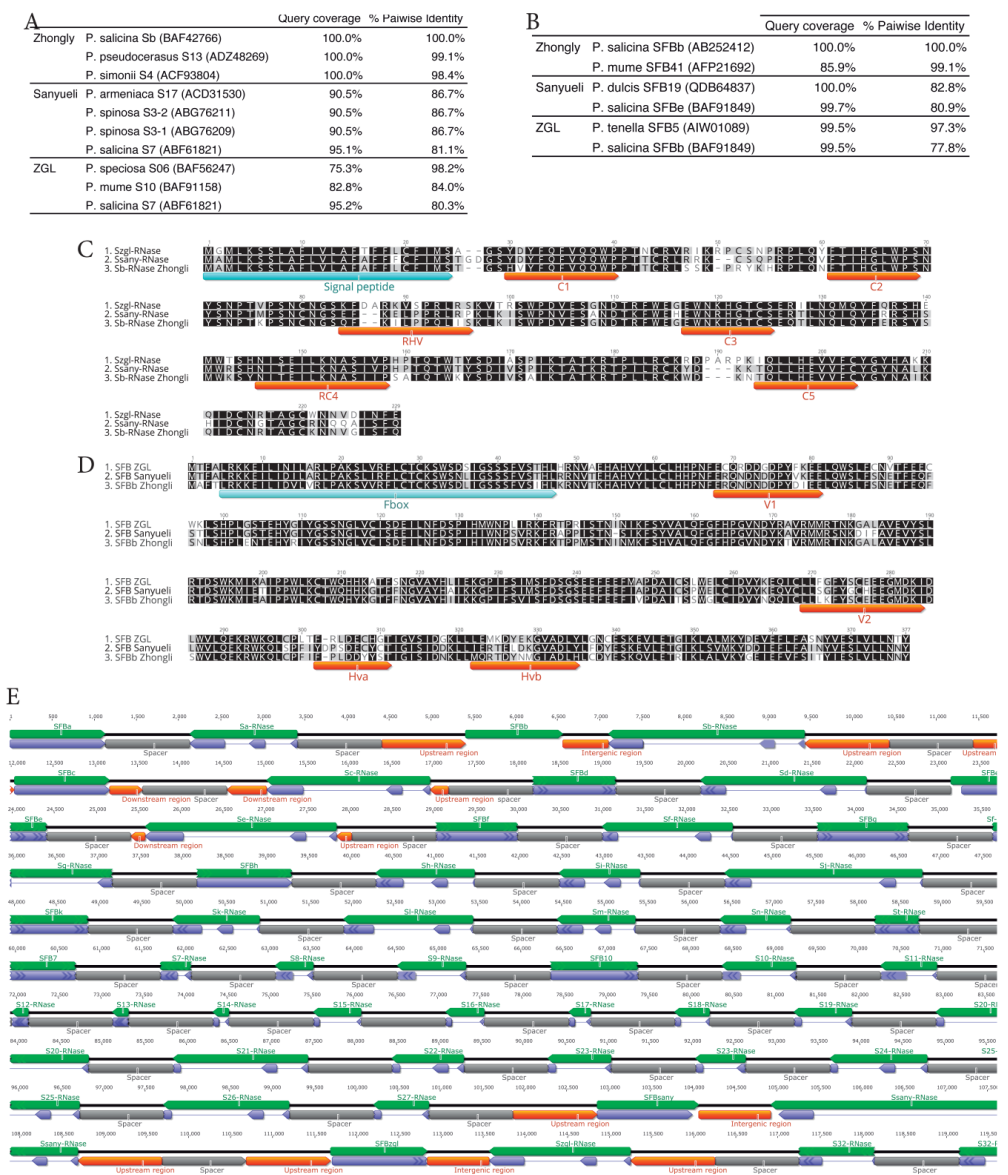
Full *S*-loci sequences retrieved from published reference genomes and synthetic reference sequence structure. (**A**,**B**) Pairwise trans-specific identities of the 6 retrieved *S-RNase* and *SFB* alleles. (**C**) Comparison of the amino-acids predicted from the coding regions of the three S-RNases. Signal peptide, conserved regions, and hypervariable regions (as defined by [66]) are highlighted. (**D**) Comparison of the amino acids predicted from the coding regions of the three SFBs. F-box, variable, and hypervariable regions (as defined by [47]) are highlighted. The amino acids of the S-RNases and SFBs were aligned using Muscle5 [67]. (**E**) Structure of the synthetic reference sequence used for aligning resequenced individuals. Genes (green), CDSs (blue), upstream, intergenic, and downstream regions (orange), and 1000 bp spacers (grey) and their orientation are illustrated. Sizes of the boxes are proportional to sequence length. Accession numbers of the sequences of each allele are given in Table 1. Full *S*-loci regions for *S_b_*, *S_sany_*, and *S_zgl_*, including 1000 bp upstream and downstream the locus and the intergenic regions, were retrieved from the threes published reference genomes of ‘Zhongly’ (Chr6:14567566:14573591), ‘Sanyueli’ (Chr6:33823425:33831243) and ZGL (Chr2:2766997:2772560), respectively.

To complete our search for all available information and, if possible, to retrieve full *S*-loci sequences, we performed a BLASTN search against the three available reference genomes with either short and conserved regions or full *S-RNase* and *SFB* sequences. In addition, we searched for highly similar candidate genes that fulfill a tail-to-tail orientation as in all *Prunus S*-loci, being able to retrieve three full *S*-loci. The *S-RNase* and *SFB* from cultivar ‘Zhongli nº6′ turned out to be 100% identical to *Sb-RNase* and *SFBb* of *P. salicina*, respectively (Figure 1A,B). All other alleles did not appear to match any sequenced allele and were therefore considered novel alleles (named *S_sany_* and *S_zgl_*). The alleles recovered from the ‘Sanyueli’ reference genome showed lower than expected trans-specific similarity within *Prunus*, whereas the *S-RNase* and *SFB* recovered from the ‘ZGL’ reference genome were highly similar to *S06 S-RNase* from *P. speciosa* and *SFB5* from *P. tenella*, respectively. High similarity between alleles of different species compared to those of the same species—called trans-specific polymorphism—is a common phenomenon within GSI [12,38,39,40]. Sequence analysis of both genes showed typical structural arrangements and size ranges as in other *P. salicina* alleles [35,41,42,43,44,45,46]. Alignment of the three *S-RNases* revealed that they have the hypothetical signal peptide sequence (and the first intron at the junction between the signal peptide and the mature protein), the three conserved regions, and the single hyper-variable region (Figure 1C) typical of ribonucleases of the T2 family [24,39]. Alignment of the three *SFB* alleles indicated that they have the single *F-box* domain, the two variable regions, and the two hypervariable regions (Figure 1D) typical of *Prunus SFB* genes [47].

To build a synthetic reference sequence, we retained the three full *S*-loci sequences, plus 1000 bp upstream and downstream the locus to increase specificity, and the longest genomic sequence of each *S*-locus gene pair and concatenated them into a rosary-like structure with 1000 bp N spacers (Figure 1E). To have a reference sequence in single strand-like structure and mimic the structure of the *S*-locus, reverse complements of *S-RNase* sequences were concatenated in tail-to-tail orientation with *SFB* alleles within each locus. In the absence of *SFB* alleles, the *S-RNases* sequences were also reverse complemented. Alignment of shotgun sequences on available reference genomes, as used for other applications, does not produce a useful alignment at the *S*-locus region due to the extreme polymorphism that characterizes this locus [33,34]. The technical advance of the method is based on the use of the entire *S*-locus allelic series of the species—i.e., all S-RNases and SFBs—to construct a synthetic reference sequence on which the resequenced genotypes are aligned. Compared to the previously described methodology based on the use of only the *S-RNase* sequence [33], our genotyping procedure is based on the parallel calling of the four *SFB* and *S-RNase* alleles that can be found in a diploid individual, and should therefore reduce allele misidentification and result in more robust genotyping.

**Table 1:** *Prunus salicina SFB* and *S-RNase* allele sequences deposited at GenBank. Sequences used to build the concatenated artificial sequence are indicated.

Allele	Cultivar (*S*-locus Genotype)	Nucleotide Sequence		Protein Sequence		Coding Sequence (CDS)	Reference
		ID	Length	ID	Length		
*SFBa*	Burmosa (*S_a_S_b_*)	AM746961.1	992 bp	CAN90151.1	331 aa	partial	[40]
*SFBa*	Ozarkpremier (*S_a_S_f_*)	DQ849087.1	977 bp	ABI15333.1	325 aa	partial	[45]
*SFBa* ^1^	Sordum (*S_a_S_b_*)	AB252410.1	1131 bp	BAF42763.1	376 aa	complete	[48]
*Sa-RNase* ^1^	Sordum (*S_a_S_b_*)	AB252411.1	1277 bp	BAF42764.1	226 aa	complete	[48]
*S1-RNase*	Red-beaut (*S_a_S_b_*)	AF433649.1	576 bp	AAP97311.1	95 aa	partial	[49]
*SFBb*	Black Golden	KJ396620.1	1131 bp	AHX39360.1	376 aa	complete	[50]
*SFBb*	Gaixiandali (*S_b_S_d_*)	DQ849088.1	978 bp	ABI15334.1	326 aa	partial	[45]
*SFBb*	Hamra Bedri	KJ396618.1	1131 bp	AHX39358.1	376 aa	complete	[50]
*SFBb*	Santa Rosa (*S_c_S_e_*)	KJ396607.1 ^2^	1131 bp	AHX39347.1	376 aa	complete	[50]
*SFBb* ^1^	Sordum (*S_a_S_b_*)	AB252412.1	1131 bp	BAF42765.1	376 aa	complete	[48]
*Sb-RNase* ^1^	Sordum (*S_a_S_b_*)	AB252413.1	2332 bp	BAF42766.1	221 aa	complete	[48]
*Sb-RNase*	Unknown	DQ646488.1	2406 bp	ABG36934.1	159 aa	partial	[51]
*SFBc*	Ain Torkia	KJ396613.1	1128 bp	AHX39353.1	375 aa	complete	[50]
*SFBc*	Bedri1 (*S_e_S_h_*)	KJ396608.1	1128 bp	AHX39348.1	375 aa	complete	[43,50,52]
*SFBc*	Meiguili (*S_c_S_e_*)	DQ849084.1	1128 bp	ABI15330.1	375 aa	complete	[47]
*SFBc* ^1^	Santa Rosa (*S_c_S_e_*)	AB280792.1	2124 bp	BAF91847.1	375 aa	complete	[53]
*Sc-RNase*	Oishiwasesumomo (*S_c_S_d_*)	AB084144.1	1781 bp	BAC20940.1	177 aa	partial	[41]
*Sc-RNase*	Santa Rosa (*S_c_S_e_*)	AB280791.1	2630 bp	BAF91846.1	230 aa	complete	[53]
*Sc-RNase*	Unknown	DQ646489.1	1920 bp	ABG36936.1	172 aa	partial	[51]
*S4-RNase*	Royal Zee (*S_c_S_e_*)	AF432418.1	1283 bp	AAP97308.1	95 aa	partial	[49]
*SFBd* ^1^	Formosa (*S_b_S_d_*)	AM746962.1	992 bp	CAN90152.1	331 aa	partial	[40]
*Sd-RNase* ^1^	Oishiwasesumomo (*S_c_S_d_*)	AB084145.1	1976 bp	BAC20941.1	169 aa	partial	[41]
*SFBe*	Aouina Hamra Bedria (*S_e_S?*)	KJ396612.1	1131 bp	AHX39352.1	376 aa	complete	[50]
*SFBe*	Black Diamant/ Black Diamond (*S_e_S_h_*)	KJ396605.1	1128 bp	AHX39345.1	375 aa	complete	[43,50]
*SFBe*	Cidre (*S_a_S_e_*)	KJ396611.1	1128 bp	AHX39351.1	375 aa	complete	[41,50]
*SFBe*	Meiguili (*S_c_S_e_*)	DQ849086.1	1036 bp	ABI15332.1	345 aa	partial	[45]
*SFBe*	Santa Rosa (*S_c_S_e_*)	AB280794.1	1248 bp	BAF91849.1	375 aa	partial	[53]
*SFBe*	Stanley	KJ396606.1 ^3^	1128 bp	AHX39346.1	375 aa	complete	[50]
*SFBe* ^1^	Unknown	DQ646490.1	1749 bp	ABG36937.1	373 aa	complete	[51]
*Se-RNase* ^1^	Santa Rosa (*S_c_S_e_*)	AB280793.1	2622 bp	BAF91848.1	239 aa	complete	[53]
*S5-RNase*	Royal Zee (*S_c_S_e_*)	AF433647.1	1553 bp	AAP97309.1	95 aa	partial	[49]
*SFBf* ^1^	Huangpili (*S*_7_*S_f_*)	DQ849089.1	972 bp	ABI15335.1	324 aa	partial	[45]
*SFBf*	Janha	KJ396610.1	996 bp	AHX39350.1	331 aa	complete	[50]
*SFBf*	Meski Hamra	KJ396614.1	1086 bp	AHX39354.1	361 aa	complete	[50]
*SFBf*	Meski Kahla	KJ396617.1	1086 bp	AHX39357.1	361 aa	complete	[50]
*SFBf*	Unknown	DQ989578.1	1081 bp	ABM54900.1	360 aa	partial	[49]
*SFBf*	Zaghwenia (*S_c_S_f_*)	KJ396615.1	993 bp	AHX39355.1	330 aa	complete	[50,52]
*Sf-RNase* ^1^	White Plum (*S_f_S_g_*)	AB084147.1	1554 bp	BAC20943.1	132 aa	partial	[41]
*Sf-RNase mRNA*	Huangpili (*S*_7_*S_f_*)	DQ512911.1	762 bp	ABF61820.1	215 aa	partial	[54]
*S6-RNase*	Wikson	AF433648.1	1212 bp	AAP97310.1	95 aa	partial	[49]
*SFBg*	Bonnie (*S_g_S_h_*)	AM746963.1	992 bp	CAN90153.1	331 aa	partial	[40]
*SFBg* ^1^	Unknown	DQ989579.1	1084 bp	ABM54901.1	361 aa	partial	[51]
*Sg-RNase* ^1^	Bonnie (*S_g_S_h_*)	AM746950.1	1536 bp	CAN90140.1	169 aa	partial	[40]
*Sg-RNase*	Honey Rosa (*S_b_S_g_*)	AB093131.1	1266 bp	BAC75456.1	79 aa	partial	[41]
*SFBh*	Ain Tasstouria	KJ396616.1	1125 bp	AHX39356.1	374 aa	complete	[50]
*SFBh*	Bedri2	KJ396619.1	1125 bp	AHX39359.1	374 aa	complete	[50]
*SFBh*	Jabounia Safra	KJ396609.1	1125 bp	AHX39349.1	374 aa	complete	[50]
*SFBh* ^1^	Nvgelei (*S_c_S_h_*)	DQ849118.1	1131 bp	ABI15337.1	376 aa	complete	[45]
*SFBh*	Unspecified	DQ646491.1	1253 bp	ABG36938.1	374 aa	partial	[51]
*Sh-RNase* ^1^	Kelsey (*S_f_S_h_*)	AB084148.1	1172 bp	BAC20944.1	175 aa	partial	[41]
*Si-RNase* ^1^	Bakemonosumomo (*S_b_S_i_*)	AB084149.1	968 bp	BAC20945.1	170 aa	partial	[41]
*Sj-RNase* ^1^	Tecumseh (*S_f_S_j_*)	AB093132.1	2346 bp	BAC75457.1	173 aa	partial	[41]
*SFBk* ^1^	Wickson (*S_f_S_k_*)	DQ992485.1	1083 bp	ABM54902.1	361 aa	partial	[44,51]
*Sk-RNase*	Friar (*S_h_S_k_*)	DQ790372.1	374 bp	ABH07013.1	63 aa	partial	[55]
*Sk-RNase* ^1^	Starkgold (*S_g_S_k_*)	AB093133.1	1035 bp	BAC75458.1	187 aa	partial	[41,56]
*Sk-RNase*	Wickson (*S_f_S_k_*)	EU113311.1	632 bp	ABW86860.1	149 aa	partial	[44,57]
*S3-RNase*	Unspecified	AF432417.1	467 bp	AAP97307.1	95 aa	partial	[58]
*Sl-RNase* ^1^	Combination (*S_g_S_l_*)	AB093134	1533 bp	BAC75459.1	188 aa	partial	[41]
*Sm-RNase* ^1^	Botan (*S_a_S_m_*)	AB093135.1	930 bp	BAC75460.1	171 aa	partial	[41]
*Sn-RNase* ^1^	Superior (*S_a_S_n_*)	AB093136	843 bp	BAC75461.1	121 aa	partial	[41,56]
*St-RNase* ^1^	Karari (*S_b_S_t_*)	AB573636.1	525 bp	BAJ13374.1	175 aa	partial	[59]
*SFB7* ^1^	Huangpili (*S*_7_*S_f_*)	DQ849085	975 bp	ABI15331.1	325 aa	partial	[45]
*S7-RNase* ^1^	Huangpili (*S*_7_*S_f_*)	AY781290.1	369 bp	AAV34703.1	94 aa	partial	[60]
*S7-RNase mRNA*	Huangpili (*S*_7_*S_f_*)	DQ512912.1	761 bp	ABF61821.1	217 aa	partial	[51]
*S8-RNase* ^1^	82-1-109	AY902455.1	450 bp	AAW83158.1	94 aa	partial	[60]
*S8-RNase mRNA*	82-1-109	DQ512913.1	750 bp	ABF61822.1	213 aa	partial	[51]
*S9-RNase* ^1^	Tai Ping Guo	AY996051.1	815 bp	AAY15124.1	94 aa	partial	[60]
*SFB10* ^1^	Mali (*S*_10_*S*_14_)	DQ849090.1	1041 bp	ABI15336.1	347 aa	partial	[45]
*S10-RNase* ^1^	Hong Men Ling	DQ003310.1	880 bp	AAX98270.1	94 aa	partial	[60]
*S11-RNase* ^1^	Zhushali	DQ512908.1	670 bp	ABF61817.1	119 aa	partial	[51]
*S12-RNase* ^1^	Kongquedan (*S*_12_*S*_13_)	DQ512909.1	197 bp	ABF61818.1	65 aa	partial	[51]
*S13-RNase* ^1^	Kongquedan (*S*_12_*S*_13_)	DQ512910.1	191 bp	ABF61819.1	63 aa	partial	[51]
*S14-RNase* ^1^	Mali (*S*_10_*S*_14_)	EF177345.1	194 bp	ABM53475.1	37 aa	partial	[45]
*S15-RNase* ^1^	Kuandiandali (*S*_15_*S*_16_)	EF177346.1	571 bp	ABM53476.1	39 aa	partial	[61]
*S16-RNase* ^1^	Kuandiandali (*S*_15_*S*_16_)	EF177347.1	460 bp	ABM53477.1	39 aa	partial	[61]
*S17-RNase* ^1^	Xinjiangsanhao (*S*_17_*S*_19_)	EF177348.1	261 bp	ABM53478.1	39 aa	partial	[61]
*S18-RNase* ^1^	Danchengxingmei (*S*_18_*S*_20_)	EF177349.1	413 bp	ABM53479.1	38 aa	partial	[61]
*S19-RNase* ^1^	Xinjiangsanhao (*S*_17_*S*_19_)	EU113259.1	690 bp	ABW88922.1	55 aa	partial	[62]
*S20-RNase* ^1^	Danchengxingmei (*S*_18_*S*_20_)	EU113260.1	1925 bp	ABW88923.1	55 aa	partial	[62]
*S21-RNase* ^1^	Zhenzhuli (*S*_21_*S*_22_)	EU113261.1	1601 bp	ABW88924.1	116 aa	partial	[62]
*S22-RNase* ^1^	Zhenzhuli (*S*_21_*S*_22_)	EU113262.1	853 bp	ABW88925.1	117 aa	partial	[62]
*S23-RNase* ^1^	Dameigui (*S*_23_*S*_24_)	EU113263.1	750 bp	ABW88926.1	54 aa	partial	[62]
*S23-RNase* ^1^	Guangdonghongli	FJ377732.1 ^4^	597 bp	ACI88749.1	64 aa	partial	[63]
*S24-RNase* ^1^	Dameigui (*S*_23_*S*_24_)	EU113264.1	1155 bp	ABW88927.1	117 aa	partial	[62]
*S25-RNase* ^1^	Unspecified	EU113265.1	920 bp	ABW88928.1	116 aa	partial	[62]
*S26-RNase* ^1^	Unspecified	EU113266.1	1492 bp	ABW88929.1	117 aa	partial	[62]
*S27-RNase* ^1^	Unspecified	EU113267.1	657 bp	ABW88930.1	54 aa	partial	[62]
*S32-RNase* ^1^	Guali	GU574195.1 ^5^	898 bp	ADD20972.1	100 aa	partial	[64]
*S41-RNase* ^1^	Qingzhouli	GU968758.1	719 bp	ADF42685.1	121 aa	partial	[65]

^1^ Used for genotyping. ^2^ BLASTN sequence is 100% identical to SFBb but the cultivar is likely wrong; ‘Santa Rosa’ was genotyped elsewhere and here as *ScSe*. ^3^ BLASTN sequence is 100% identical to *SFBe* but the cultivar name is reported in the literature as *Prunus domestica*. ^4^ A duplicate case: same name given to different alleles. ^5^ Highly similar to *S10-Rnases* at the nucleotide level.

### 2.2. A Powerful S-Allele Calling Procedure

**Figure 2:**
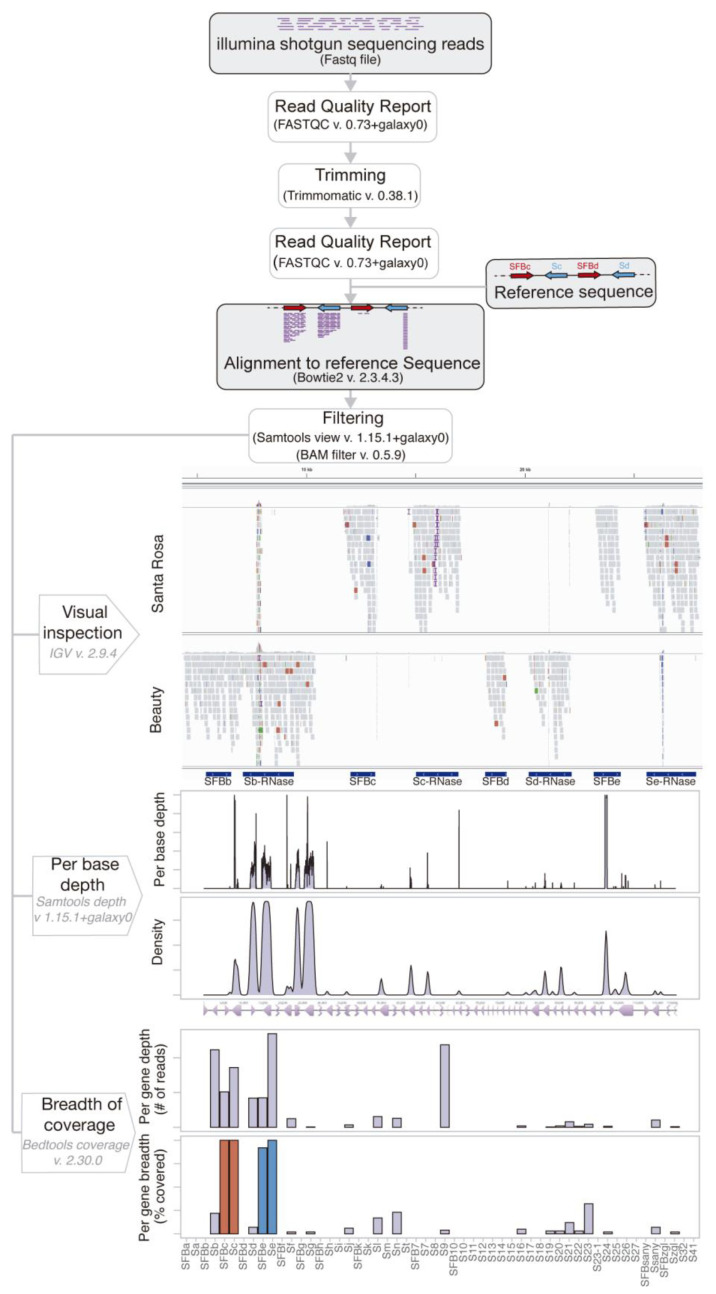
Bioinformatics pipeline for *S*-genotyping by high throughput sequencing. The visual inspection illustrates cultivars ‘Santa Rosa’ and ‘Beauty; the per base depth and per gene depth of the coverage illustrate the cultivar ‘Santa Rosa’ (see Figure S1 for all genotypes analyzed).

Raw individual shotgun sequences from 88 Japanese plum cultivar samples (83 genomic, four exon targeted-capture, and one transcriptome libraries (Table S1 and references therein) were aligned to the synthetic sequence using Bowtie2, following the end-to-end default sensitive mode (Figure 2). To increase the stringency of the alignment for higher *S*-allele specificity, reads with a mapping quality lower than 20, reads shorter than 20bp in length, and reads with more than two mismatches with the reference sequence were filtered out.

To accurately genotype the *S*-locus of all samples, we followed a strategy that combined a visual inspection of the alignment with the implementation of a quantitative analysis. First, we visually inspected all alignments and established a preliminary *S*-genotype based on gene coverage. Second, we used different tools that report per-base depth and per-gene breadth of the coverage. The per-base depth was plotted as raw data (area graph) and fitted as probability density distribution. The per-gene breadth of the coverage was plotted as bar plots of the number of reads per gene and the proportion of the gene covered by at least one read base (see Figure S1 for all genotypes analyzed). Whereas each plot showed a bit of information about the likely *S*-genotype, careful examination of all figures followed by quantitative analysis was necessary to contrast the preliminary visual inspection and to call a positive *S*-allele. The probability density plot was especially informative about the actual likelihood to observe a given peak. The size of the area below the curve that a given gene covers is in fact and approximation of the probability of observing that peak considering that the whole area under the curve should sum up to one (see Table S2 for all genotypes analyzed). The proportion of the gene covered by at least one read base was especially relevant for discarding those unspecific alignments with high coverage but limited breadth, which are characteristic of intronic and intergenic regions with repetitive and transposable elements that are frequently found throughout the genome. We considered adding the rest of the genome to the synthetic sequence, but this approach significantly increased the duration of the alignment procedure with similar results to our three-steps validation procedure. Positive *S*-allele calls were based on the highest gene breadth of coverage and high likelihood in congruence with the visual inspection of the alignment.

Of the 88 shotgun samples, we were able to unambiguously genotype two *S*-alleles in 74 cultivars, only one *S*-allele in 11 cultivars, and none of the *S*-alleles in four cultivars (Table 2). The low depth and breadth of coverage in the *S*-locus regions extracted from the reference genomes (*S_b_*, *S_sany_* and *S_zgl_*, see Figure S1) for most cultivars not carrying those three alleles illustrate the high degree of polymorphism at the *S*-locus, and how reference genomes cannot be used for genotyping this locus. Our methodology clearly improved the approach used in *M. domestica* based solely on per base depth of coverage, which is highly sensitive to non-specific coverage of intronic regions [33]. Although this limitation was successfully circumvented in the previous work by setting the per-base depth of coverage only for exonic regions, this might lead to misidentification of alleles in species with lower polymorphism than apple in transcribed regions. Our approach, which includes intronic regions while solving the problem of non-specific alignment, allowed us to reduce these identifications errors.

All individuals with a putative *S*_10_ or *S*_32_ allele did not show an exclusive alignment to any of the two loci. Although this result was expected due to the highly similarity between both alleles at the nucleotide level, we finally decided to assign a mixed genotype S_10/32_ to all individuals with this genotype. De novo assembly or full sequencing of both *S*-alleles in their respective cultivars will clarify whether they represent the same or different alleles. All unidentified *S*-alleles were assumed to be novel unsequenced alleles. Furthermore, since the *S-RNase* and *SFB* genes are known to be expressed in flower tissues during the anthesis period [7,8,9,10], we demonstrated that the *S*-genotype of the cultivar ‘Sanyueli’, as established from genomic reads (S_10/32_S_sany_) could also be inferred from flower transcriptome reads (Figure S1, Table S2). The lower likelihood of positive calls and shorter breadths of gene coverage can be explained by the absence of coverage within the intronic regions. In addition, we extended our analysis to test whether shotgun sequences from exome-targeted-capture libraries could be useful for *S*-genotyping. We chose four libraries from cultivars of previously known genotypes [35,41,42] and aligned them to the synthetic sequence. Although we successfully confirmed the *S*-genotype of all 4 cultivars (Table 2), lower values of likelihood and breadth of coverage should also reflect the absence of intronic coverage among others (Figure S1, Table S2). In summary, our approach represents a relevant addition to the methodologies previously described in *M. domestica* [33] and *Arabidopsis halleri* [34], as it combined the strategy of using the entire *S*-locus, the high stringency of the alignment of shotgun sequences from different types of libraries, and the quantitative analysis based on the determination of both the probability of positive calls and the percentage of gene coverage.

### 2.3. S-Allele Genotypes, Methodology Validation, and Incompatibility Groups

The approach developed here allowed for the identification of the *S*-genotype in 88 Japanese plum cultivars, 74 of them reported for the first time (Table 2). At least two *S*-alleles were identified in 74 cultivars, which, according to their *S*-allele composition, were assigned to 22 incompatibility groups, including nine new incompatibility groups reported for the first time (XXVII-XXXV, Table 2). In addition, 18 cultivars were included in group 0, since no other cultivars with the same *S*-genotype have been reported so far and, therefore, they can be considered as potential universal pollinators [14]. This information could be highly valuable for growers and breeders, since the knowledge of incompatibility relationships is intended to help fruit growers to select compatible pollinizers and breeders to choose parental genotypes.

To validate our methodology, we compared our results with already known *S*-genotypes of those cultivars for which *S*-locus sequences are available in the GenBank (Table 1 and Table 2). The results confirmed the *S*-genotype of 13 cultivars: ‘Sordum’ [35], ‘Oishiwase’ and ‘Taiyo’ [41], ‘Angeleno’ [43], ‘Fortune’ and ‘Friar’ [68], ‘Jinshali’ and ‘Qiuji’ [45], ‘Pingguoli’ and ‘Zuili’ [46], and the two self-compatible cultivars ‘Honeyrosa’ [42] and ‘Santa Rosa’ [68]. However, the *S*-genotype of ‘Beauty’ (*S_b_S_d_*) and ‘Friar’ (*S_b_S_h_*) differed from previously reported genotypes. ‘Beauty’ was previously genotyped as *S_c_S_e_* [41] and ‘Friar’ as *S_h_S_k_* [55]. Based on a previous report on the *S*-allele inheritance in ‘Friar’ [68], its most likely *S*-genotype is *S_b_S_h_*. On the other hand, further research using additional plant material from ‘Beauty’ would be needed to clarify its *S*-genotype. Although further validation of the remaining cultivars -as more *S*-locus sequences become available- and clarification of those conflicting results would provide definitive support for the validity of this methodology, confirmation of the *S*-genotype in 14 cultivars is a strong indication that our methodology is reliable.

In 11 cultivars, only one *S*-allele could be identified and, therefore, they were not assigned to any incompatibility group and were considered unclassified (Table 2). In four cultivars, no *S*-allele could be identified, suggesting that at least two novel *S*-alleles were detected. *S*-allele de novo assembly complemented with PCR analysis based on the amplification of *S*-*RNase* and *SFB* genes in these 15 unclassified cultivars would be needed to identify the new *S*-alleles and clarify their *S*-genotype. Three pairs of cultivars have a highly similar names and the same *S*-genotype (‘Changlixiangjiaoli’ and ‘Changlixiangbian’, ‘Guofeng2’ and ‘N2 Guofeng’, ‘Cuihongli’ and ‘Cuihong’) so they could be considered synonyms. On the other hand, ‘Li he’ and ‘Lihe’, with highly similar names, showed different *S*-genotypes, so they are distinct genotypes and could be considered homonyms. The presence of three and four alleles in the cultivars ‘Guiyang’ and ‘Xingyikongxinli’, respectively, means that either they have undergone some *S*-locus duplication events, or, more likely, they are triploid and tetraploid individuals, respectively.

No information on self-(in)compatibility is available for most of the cultivars tested (all except two self-compatible cultivars ’Honeyrosa’ [42] and ‘Santa Rosa’ [68]). Controlled pollinations and subsequent microscopic analysis of pollen tube growth in self-pollinated flowers would be necessary, since the identification of the *S*-alleles of a cultivar does not accurately predict its self-(in)compatibility [14].

Japanese plum, which include pure *P. salicina* cultivars and hybrids of *P. salicina* with other *Prunus* species [14,69], is an ideal system to undertake such *S*-genotyping for several scientific and practical reasons. *P. salicina* is considered to have originated in south-western China from where it spread to other Chinese territories, then to Japan, from Japan to California, and from there to other parts of the world such as Europe, New Zealand, South America and South Africa [70,71,72]. The highest diversity of *S*-alleles was observed in cultivars putatively originating from the southern provinces of China (Table 2, Table S1), further supporting the putative origin of the species in this region. Furthermore, no cultivar of putative Chinese origin had the *S_a_* and *S_l_* alleles, suggesting a probable introgression of these alleles from other *Prunus* species through hybridization. Interestingly, the cultivars ‘Changlixiangjiaoli’ and ‘Changlixiangbian’ from Hebei province in China, genotyped here as *S_e_S_k_* (Table 2 and Table S1), were claimed to belong to *P. simonii* in a previous study aimed at characterizing the origin and diversity of this species [72]. Whether this *S*-genotype reflects trans-specific polymorphism or an introgression of one or both alleles in *Japanese plum* hybrids is unknown. Undoubtedly, further studies on Chinese germplasm aimed at distinguishing pure *P. salicina* cultivars from interspecific hybrids will reveal more introgressed *S*-alleles.

**Table 2:** Incompatibility groups (I. G.), *S*-genotype and library type (E: exon-targeted capture, G: genomic, T: transcriptome) of 88 Japanese plum cultivars.

I. G.	*S*-alleles	Cultivar	Origin ^1^	Library	Reference
I	*S_a_S_b_*	Sordum	Japan	E	[35]
II	*S_b_S_c_*	Fortune	California, USA	G	[68]
		Zaoshengyueguang	Japan	G	
		Dashizhongsheng	Japan	G	
		Taiyo	Japan	E	[41]
III	*S_b_S_f_*	Zhenyuanqiyuexiang	Guizhou China	G	
IV	*S_b_S_h_*	Friar	California, USA	G	[68]
		Friar ^2^	California, USA	G	[55] (*S_h_S_k_*)
		Qiuji	Japan	G	[45]
		Guofeng7	Liaoning, China	G	
		C20	Spain	G	
		Zhongly nº 6	Northern China	G	
V	*S_b_S_i_*	Fujianfurongli	Fujian, China	G	
VI	*S_f_S_h_*	Satsuma	California, USA	G	
VII	*S_c_S_h_*	Fulihong	Shaanxi, China	G	
		Angeleno	California, USA	G	[43]
IX	*S_f_S_g_*	Jinshali	Yunnan, China	G	[45]
		Cehengjixueli	Guizhou, China	G	
XIV	*S_a_S_c_*	C46	Spain	G	
XIX	*S_b_S_d_*	Abazhoumeiguili	Sichuan, China	G	
		Beauty ^2^	California, USA	G	[41] (*S_c_S_e_*)
XXI	*S_e_S_k_*	Wuxiangli	Hebei, China	G	
		Changlixiangjiaoli	Hebei, China	G	
		Changlixiangbian	Hebei, China	G	
XXIII	*S_c_S_d_*	Dashizaosheng	Japan	G	
		Jingshang	Japan	G	
		Oishiwase	Japan	G	[41]
		Oishiwase	Japan	E	[41]
XXIV	*S_e_S*_11_	Jinxiqiuli	Liaoning, China	G	
		Qiyuexiang	Liaoning, China	G	
XXV	*S*_15_ *S*_16_	Pingguoli	Liaoning, China	G	[46]
XXVII ^3^	*S_b_S*_7_	Heyuansanhuali	Guangdong, China	G	
		Qingpihongxin	Guangdong, China	G	
		Zaohuangli	Anhui, China	G	
		Jinganzhushali	Jiangxi, China	G	
		Jaiqingzi	Jiangsu, China	G	
XXVII ^3^	*S_b_S*_8_	Huahongli	Yunnan, China	G	
		Brace	California, USA	G	
XXIX ^3^	*S_e_S*_10/32_	Shuili	Guangxi, China	G	
		Dahuili	Henan, China	G	
		Xiangjiaoli	Liaoning, China	G	
		Qiuxiang	Liaoning, China	G	
XXX ^3^	*S_g_S_k_*	Yuanshuai	Japan	G	
XXXI ^3^	*S_h_S*_10/32_	Guofeng2	Liaoning, China	G	
		N2 Guofeng	Liaoning, China	G	
XXXII ^3^	*S_i_S*_9_	Changkuangsanhuali	Guangdong, China	G	
		Sanhua plum	Guangdong, China	G	
XXXIII ^3^	*S*_10/32_ *S*_11_	Tiankanmali	Sichuan, China	G	
		Li He	China	G	
		Wushan plum	Chongging, China	G	
		Wanshuang plum	Chongging, China	G	
XXXIV ^3^	*S*_11_ *S*_20_	Yuhuangli	Shaanxi, China	G	
		Huangli	Shandong, China	G	
		Lihe	Hebei, China	G	
XXXV ^3^	*S_b_S_c_S_f_*	Guiyang	Japan	G	
Group 0	*S_a_S_l_*	Hongshou	Japan	G	
	*S_b_S*_11_	Yanzhili	Fujian, China	G	
	*S_d_S_g_*	Fenghuali	Zhejiang, China	G	
	*S_e_S*_12_	Huangguli	Zhejiang, China	G	
	*S_e_S*_20_	Daqingke	Shandong, China	G	
	*S_h_S*_15_	Guofeng17	Liaoning, China	G	
	*S_h_S*_7_	Zuili	Zhejiang, China	G	[46]
	*S_k_S*_8_	Bullbank	California, USA	G	
	*S*_7_ *S*_20_	Pingdingxiang	Shandong, China	G	
	*S*_8_ *S*_9_	Wanshuhuanai	Fujian, China	G	
	*S*_10/32_ *S*_15_	Zhengzhouzaoli	Henan, China	G	
	*S*_10/32_ *S_sany_*	Sanyueli	Guangdong, China	G	
		Sanyueli	Guangdong, China	T	
	*S*_10/32_ *S_zgl_*	Cuihongli	Sichuan, China	G	
		Cuihong plum	Sichuan, China	G	
	*S*_11_ *S*_16_	Saozouli	Guangxi, China	G	
	*S_e_S_k_S*_10/32_ *S*_11_	Xingyikongxinli	Guizhou, China	G	
Cultivars in which only one *S* allele could be identified					
	*S_b_*	Cuipinwannai	Fujian, China	G	
	*S_c_*	Guoli	Liaoning, China	G	
	*S_e_*	Tianmumili	Zhejiang, China	G	
	*S_e_*	Hongxinli	Anhui, China	G	
	*S_h_*	Guomei	Liaoning, China	G	
	*S*_10/32_	Yinhong plum	Sichuan, China	G	
	*S*_10/32_	Fengtang plum	Guizhou, China	G	
	*S*_11_	Tongkeli	Guangxi, China	G	
	*S*_19_	Non-specified	Tibet, China	G	
	*S*_21_	Damili	Guangdong, China	G	
	*S*_22_	Zhushali	Yunnan, China	G	
Cultivars in which no *S* allele could be identified					
		Kuaishili	Guangdong, China	G	
		Lushanli	Jiangxi, China	G	
		Ni Ma Qu Ji Li	China	G	
		Wuyuecui	Sichuan, China	G	
Self-compatible cultivars					
SC	*S_b_S_g_*	Honeyrosa	Japan	E	[42]
SC	*S_c_S_e_*	Santa Rosa	California, USA	G	[68]

^1^ Presumed origin, and in its absence the sampling location is given. ^2^ Cultivars described with different *S*-genotype. ^3^ New Incompatibility Groups first reported herein.

## 3. Materials and Methods

### 3.1. Building the Reference Synthetic S-Loci Sequence

Japanese plum sequences for *S-RNase* and *SFB* were retrieved from NCBI based on the literature and on direct database search. In addition, we conducted a BLASTN search on the three available Japanese plum reference genomes of Chinese origin (cultivars ‘Sanyueli’ assembly V2.0 at rosaceae.org [73], ‘Zhongli’ assembly V1.0 at rosaceae.org [74] and ‘ZGL’ assembly GCA_020226455.1 at NCBI [75]) and recovered three full length *S*-locus regions. The synthetic sequence was built by concatenating individual sequences in a string-like structure. Depending on the sequences available per locus, it contained sequences from three full *S*-locus regions, individual *S*-locus sequences in tail-to-tail orientation (SFB is transcribed on the forward strand and S-RNase on the reverse), and individual *S-RNase* sequences for loci with unsequenced *SFB* alleles. BLAST searches were performed at NCBI [76] or locally on the online platform useglaxy.eu using the Galaxy BLAST+ blastn v 2.10.1+galaxy0 tool [77,78]. Sequence editing, alignment, and concatenation were performed with Geneious prime 202.2.2.

### 3.2. Alignment to Reference Sequence

Individual shotgun sequencing reads from Japanese plum genomic, transcriptomic and exon targeted-capture libraries were aligned to the synthetic sequence. Alignment was performed on the online platform usegalaxy.eu [78] following the pipeline outlined in Figure 2. The raw reads were first subjected to quality control (FastQC, [79]), then trimmed (Trimmomatic [80]), and finally aligned to the reference sequence using Bowtie2 [81] with the end-to-end default sensitive mode. Samtools View [82] was used to filter out reads with mapping quality below 20, and Bam Filter [83] was used to filter out reads shorter than 20 bp or having more than 2 mismatches with the reference sequence.

### 3.3. S-Allele Typing Methodology

To accurately type *S*-allele genotypes, we used a combination of visual inspection of the alignment using IGV [84] and quantitative analyses using SAMtools [82] and BEDtools coverage [85], followed by graphical analyses using the R package ggplot2 [86]. The likelihood of a positive call was inferred by integrating the probability density distribution of read depth at the gene level, and by determining the proportion of the gene covered by reads. Final *S*-allele call was based on full congruence between the three approaches. A positive call should have a high probability, the highest proportion of gene coverage, and should be congruent with visual inspection of the alignment.

## 4. Conclusions

Building on previous *S*-genotyping procedures [33,34], we developed a modified and robust methodology to *S*-genotype Japanese plum cultivars using whole genome shotgun sequences. The technical advance of the method is based on the use of the entire *S*-locus allelic series of the species to construct a synthetic reference sequence on which resequenced genotypes are aligned. *S*-genotyping is based on parallel calling of the four alleles that form the *S*-locus, combined with a simple but powerful quantitative methodology. Further work is underway to de-novo-assemble Japanese plum *S*-alleles and obtain longer sequence for many alleles for which only short sequences are available or discover novel *SFB* and *S-RNases* alleles.

*S*-genotyping cultivars of Chinese origin would ultimately provide valuable information on the original allelic series that can be found in pure *P. salicina* cultivars and would allow us to identify all those *S*-alleles, and their respective species, that are the result of the frequent hybridization events in modern Japanese plum hybrid cultivars. It would also enable us to build a comprehensive database of inter-compatibility relationships for all available germplasm, which would be of great value for breeders and growers. The approach developed here could be applied to any other plant species—with a GSI system—provided that several *S*-loci have been sequenced. A study is underway to apply this *S*-genotyping-by-sequencing procedure to several economically important *Prunus* fruit tree species such as *Prunus armeniaca*, *Prunus avium* and *Prunus dulcis*.

## Supplementary Materials

The following supporting information can be downloaded at: https://www.mdpi.com/article/10.3390/ijms24043932/s1, References [87,88,89,90,91,92,93,94,95] are cited in the Supplementary Materials.
