# Supplementary material for: S-Locus Genotyping in Japanese Plum by High Throughput Sequencing Using a Synthetic S-Loci Reference Sequence
Source: Int J Mol Sci. 2023 Feb 15;24(4):3932. doi: 10.3390/ijms24043932 (PMC9960950; doi:10.3390/ijms24043932)
Supplement: Supplementary file 1 [file ijms-24-03932-s001.zip › Figure_S1.pdf]

Figure S1. *Per Base depth and per gene breadth of the coverage for all analysed shotgun sequences*. For each cultivar analysed (aligned and filtered shotgun sequence) there are four plots. Upper left: per base depth of the coverage; lower left: probability density function fitted to the per base depth of coverage; upper right: number of reads per gene, lower right: percentage of the gene covered by at least one read base. Final positive calls and the identity of the alleles are indicated in the lower right panel. For all plots, the x-axis labels (synthetic sequence on the left and genes ids within the synthetic sequence on the right) are plotted at bottom of each page.

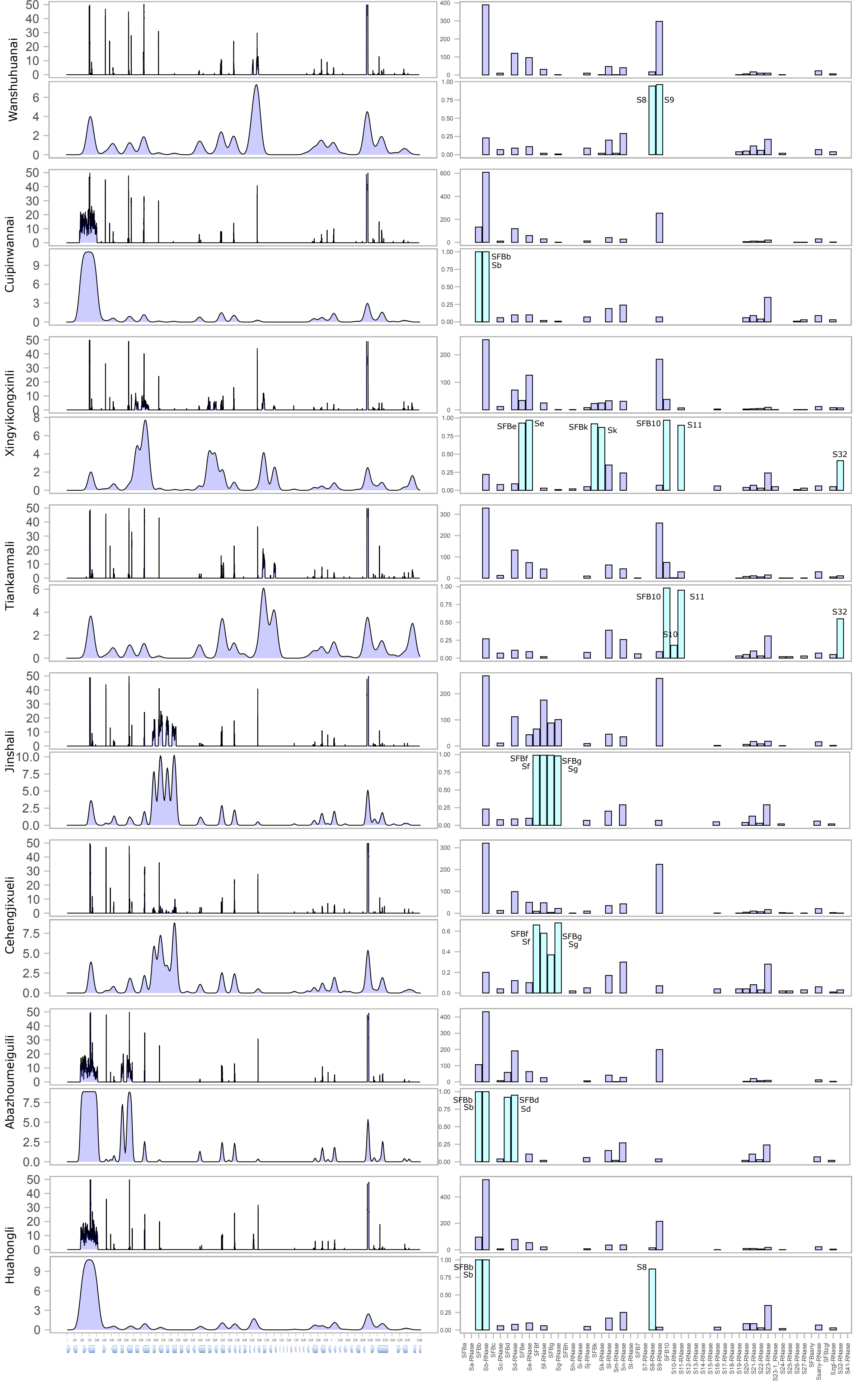

Wanshuhuanai
S8
S9
Cuipinwannai
SFBb
Sb
Xingyikongxinli
SFBe
Se
SFBk
Sk
SFB10
S11
S32
Tiankanmali
SFB10
S11
S10
S32
Jinshali
SFBf
Sf
SFBg
Sg
Cehengjixueli
SFBf
Sf
SFBg
Sg
Abazhoumeiguili
SFBb
Sb
SFBd
Sd
Huahongli
SFBb
Sb
S8
SFBa
Sa-RNase
SFBb
Sb-RNase
SFBc
Sc-RNase
SFBd
Sd-RNase
SFBe
Se-RNase
SFBf
Sf-RNase
SFBg
Sg-RNase
SFBh
Sh-RNase
Si-RNase
Sj-RNase
SFBk
Sk-RNase
Sl-RNase
Sm-RNase
Sn-RNase
St-RNase
SFB7
S7-RNase
S8-RNase
S9-RNase
SFB10
S10-RNase
S11-RNase
S12-RNase
S13-RNase
S14-RNase
S15-RNase
S16-RNase
S17-RNase
S18-RNase
S19-RNase
S20-RNase
S21-RNase
S22-RNase
S23-RNase
S23-1_RNase
S24-RNase
S25-RNase
S26-RNase
S27-RNase
SFBsany
Ssany-RNase
SFBzgl
Szgl-RNase
S32-RNase
S41-RNase

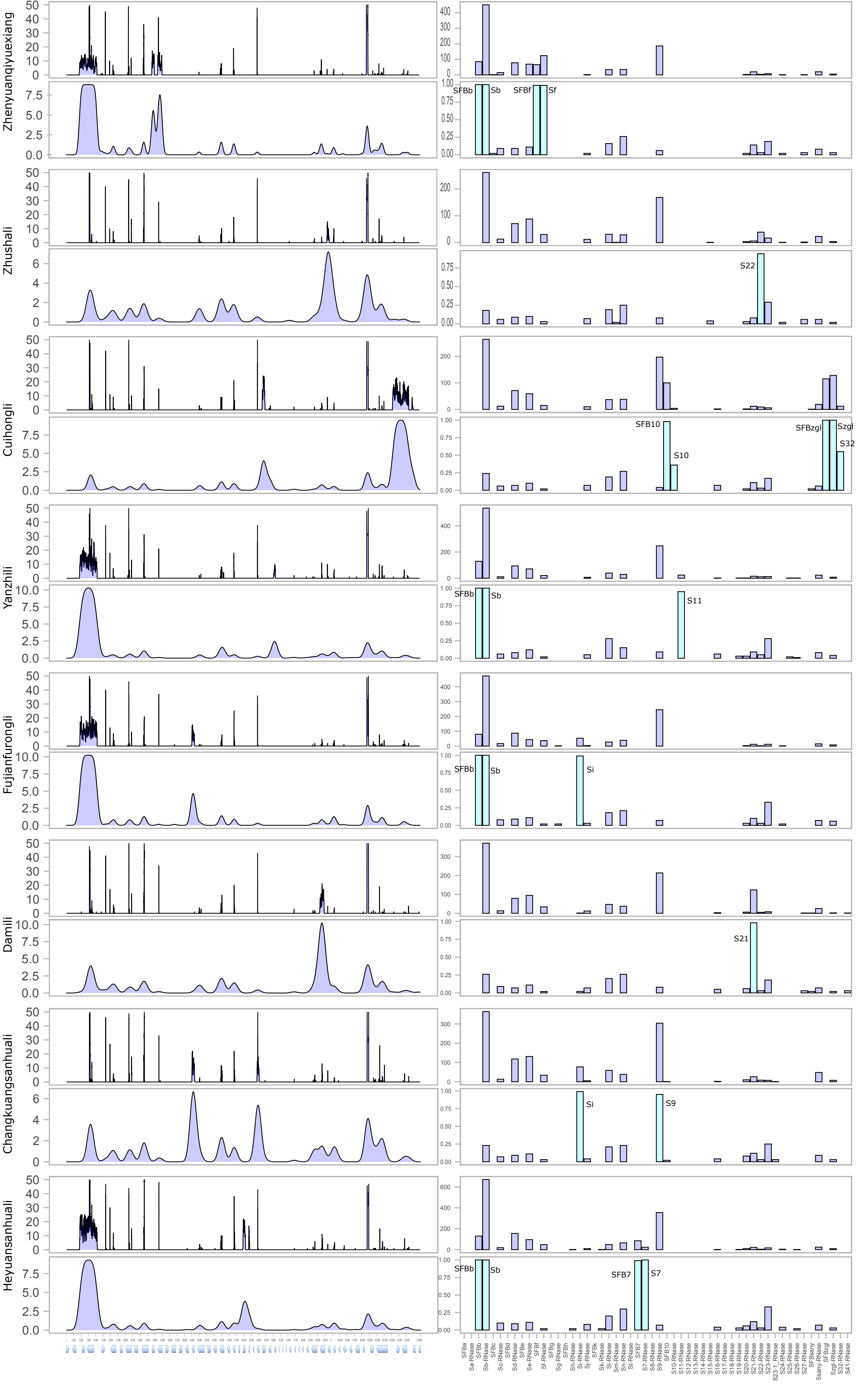
Zhenyuanqiyuexiang
SFBb
Sb
SFBf
Sf
Zhushali
S22
Cuihongli
SFB10
S10
SFBzgl
Szgl
S32
Yanzhili
SFBb
Sb
S11
Fujianfurongli
SFBb
Sb
Si
Damili
S21
Changkuangsanhuali
Si
S9
Heyuansanhuali
SFBb
Sb
SFB7
S7

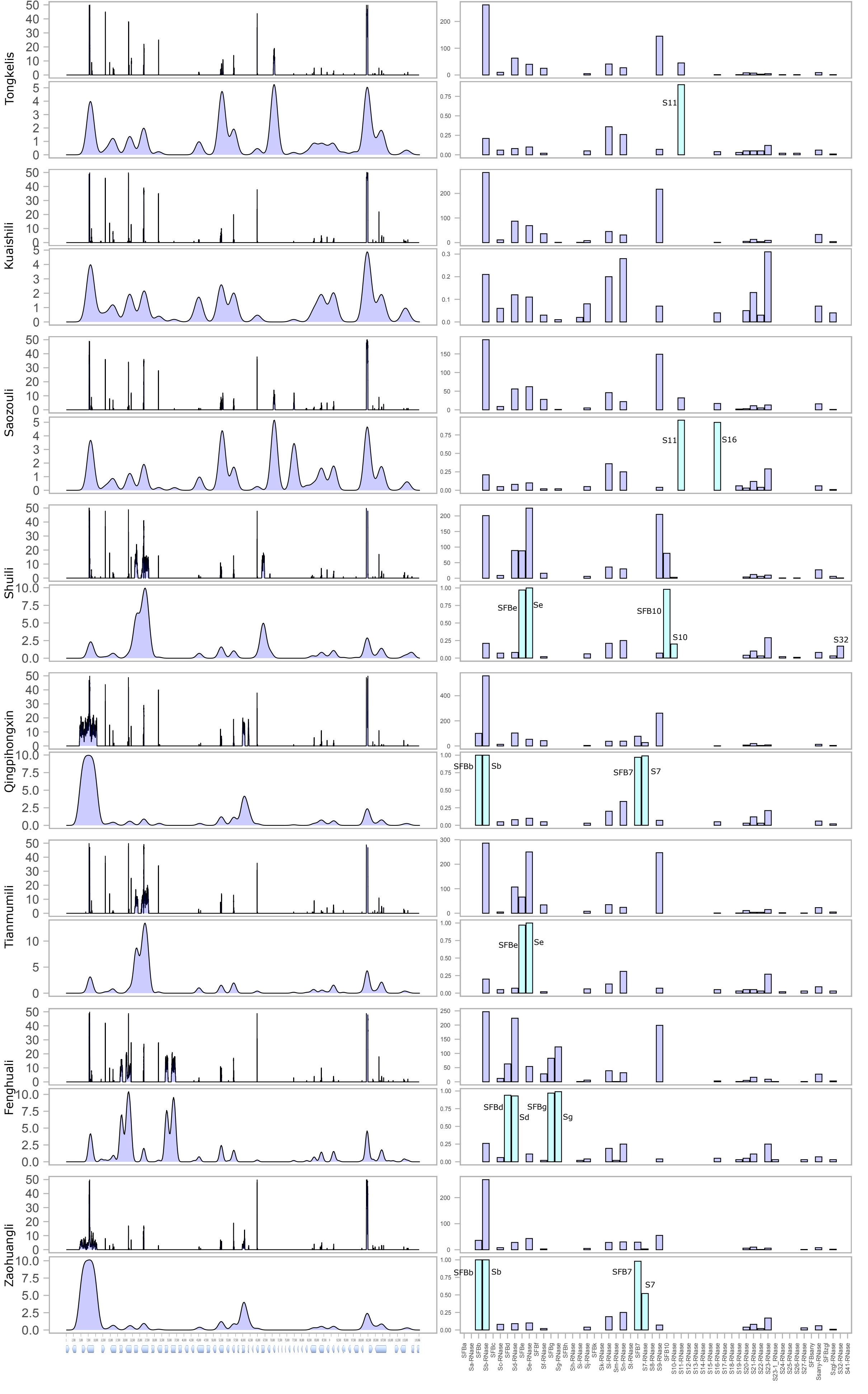
Tongkelis
Kuaishili
Saozouli
Shuili
Qingpihongxin
Tianmumili
Fenghuali
Zaohuangli
S11
S16
SFBe
Se
SFB10
S10
S32
SFBb
Sb
SFB7
S7
SFBd
Sd
SFBg
Sg
SFBa
Sa-RNase
SFBb
Sb-RNase
SFBc
Sc-RNase
SFBd
Sd-RNase
SFBe
Se-RNase
SFBf
Sf-RNase
SFBg
Sg-RNase
SFBh
Sh-RNase
Si-RNase
Sj-RNase
SFBk
Sk-RNase
Sl-RNase
Sm-RNase
Sn-RNase
St-RNase
SFB7
S7-RNase
S8-RNase
S9-RNase
SFB10
S10-RNase
S11-RNase
S12-RNase
S13-RNase
S14-RNase
S15-RNase
S16-RNase
S17-RNase
S18-RNase
S19-RNase
S20-RNase
S21-RNase
S22-RNase
S23-RNase
S23-1_RNase
S24-RNase
S25-RNase
S26-RNase
S27-RNase
SFBsany
Ssany-RNase
SFBzgl
Szgl-RNase
S32-RNase
S41-RNase

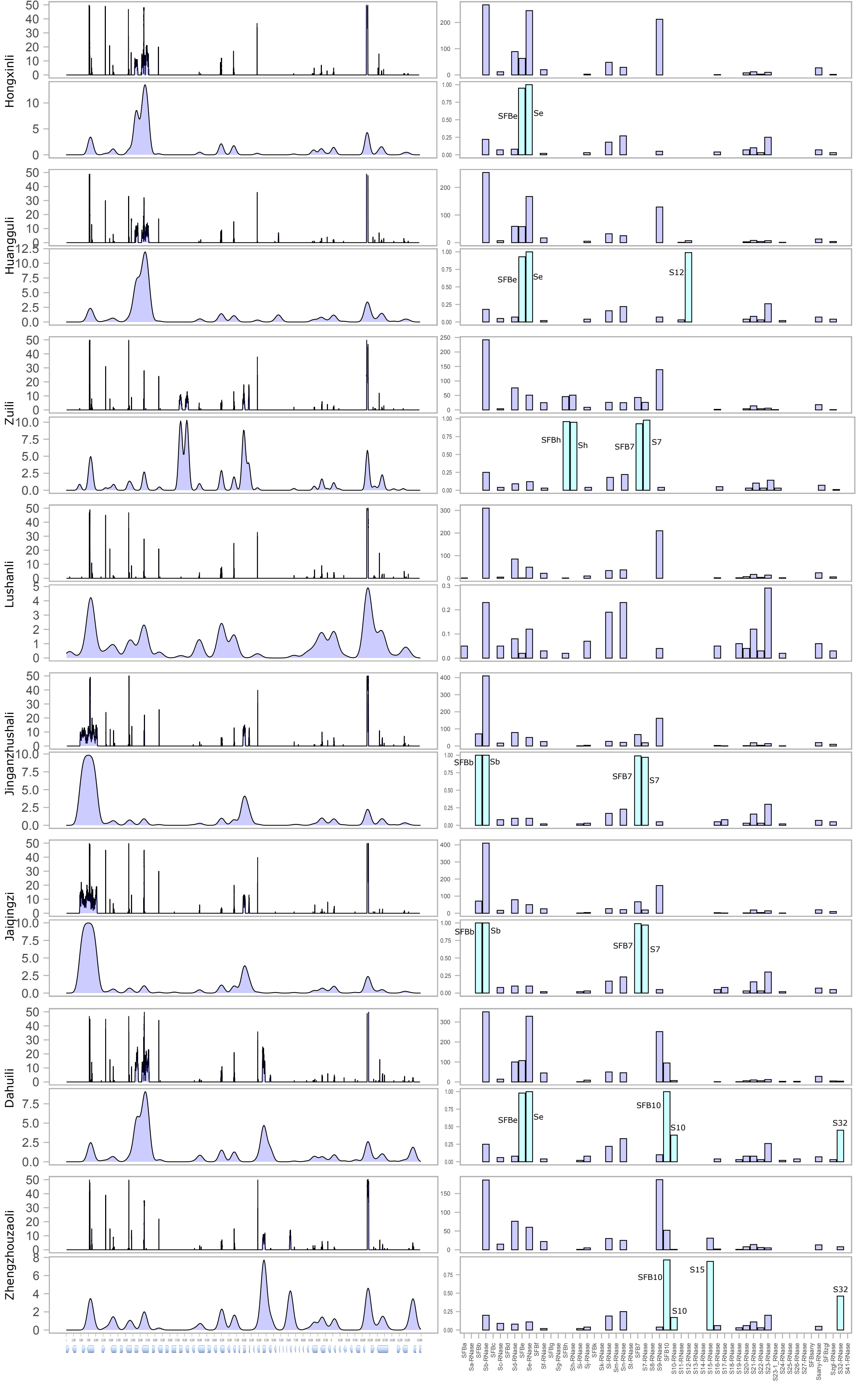
Hongxinli
Huangguli
Zuili
Lushanli
Jinganzhushali
Jaiqingzi
Dahuili
Zhengzhouzaoli
SFBe
Se
S12
SFBh
Sh
SFB7
S7
SFBb
Sb
SFB10
S10
S32
S15

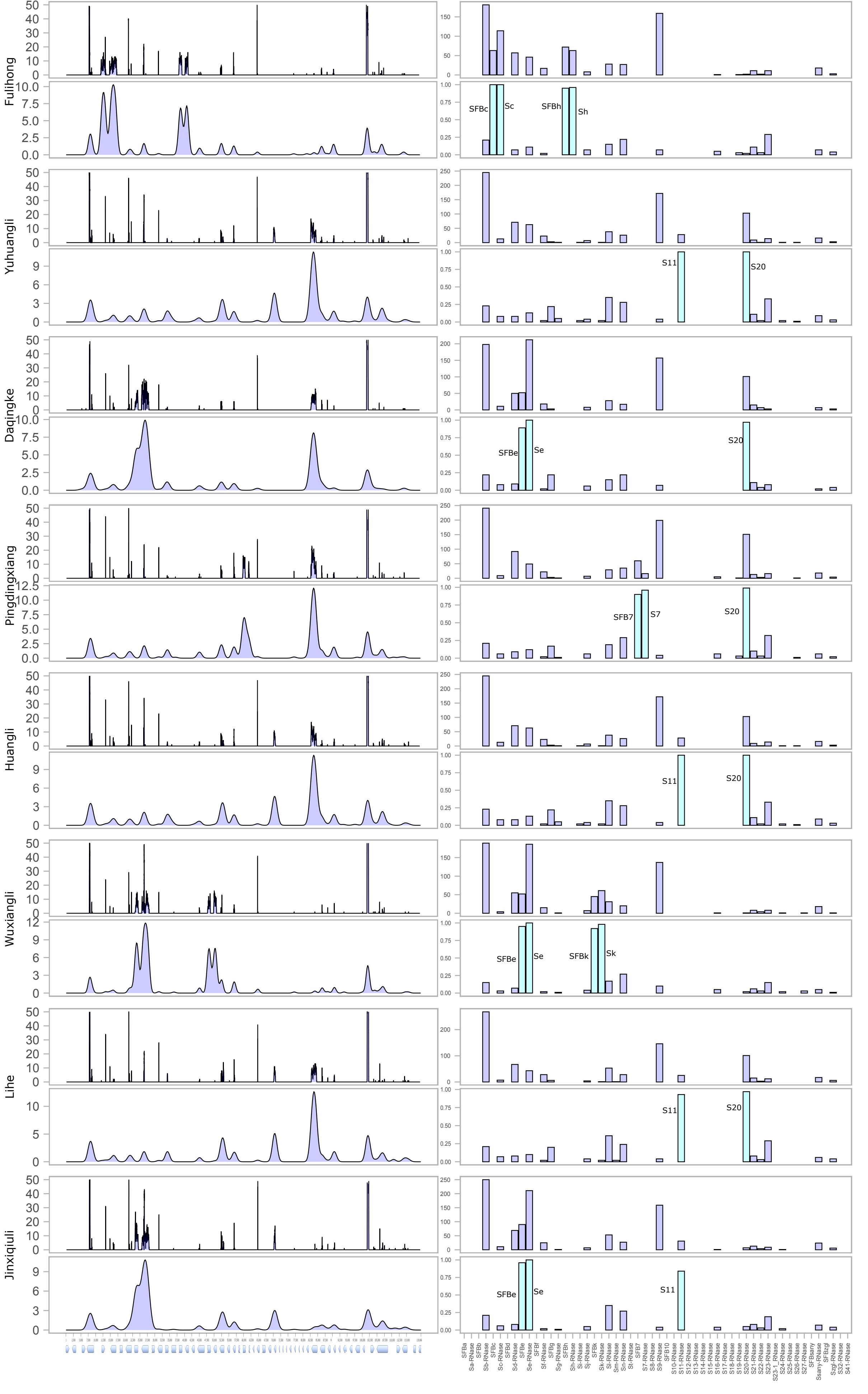
Fulihong
SFBc
Sc
SFBh
Sh
Yuhuangli
S11
S20
Daqingke
SFBe
Se
S20
Pingdingxiang
SFB7
S7
S20
Huangli
S11
S20
Wuxiangli
SFBe
Se
SFBk
Sk
Lihe
S11
S20
Jinxiqiuli
SFBe
Se
S11

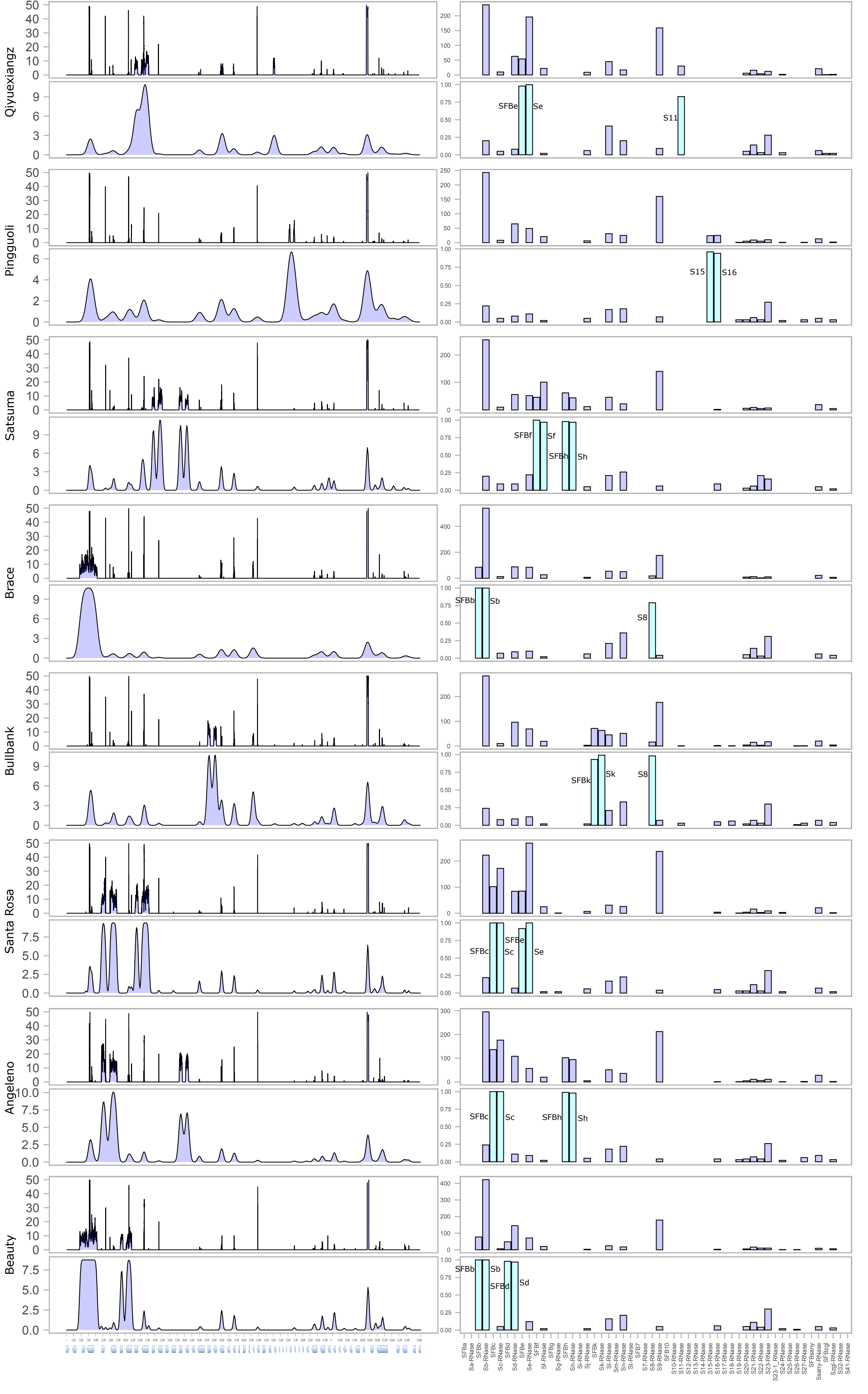
Qiyuexiangz
Pingguoli
Satsuma
Brace
Bullbank
Santa Rosa
Angeleno
Beauty
SFBe
Se
S11
S15
S16
SFBf
Sf
SFBh
Sh
SFBb
Sb
S8
SFBk
Sk
SFBc
Sc
SFBd
Sd
SFBa Sa-RNase SFBb Sb-RNase SFBc Sc-RNase SFBd Sd-RNase SFBe Se-RNase SFBf Sf-RNase SFBg Sg-RNase SFBh Sh-RNase Si-RNase Sj-RNase SFBk Sk-RNase Sl-RNase Sm-RNase Sn-RNase St-RNase SFB7 S7-RNase S8-RNase S9-RNase SFB10 S10-RNase S11-RNase S12-RNase S13-RNase S14-RNase S15-RNase S16-RNase S17-RNase S18-RNase S19-RNase S20-RNase S21-RNase S22-RNase S23-RNase S23-1_RNase S24-RNase S25-RNase S26-RNase S27-RNase SFBsany Ssany-RNase SFBzgl Szgl-RNase S32-RNase S41-RNase

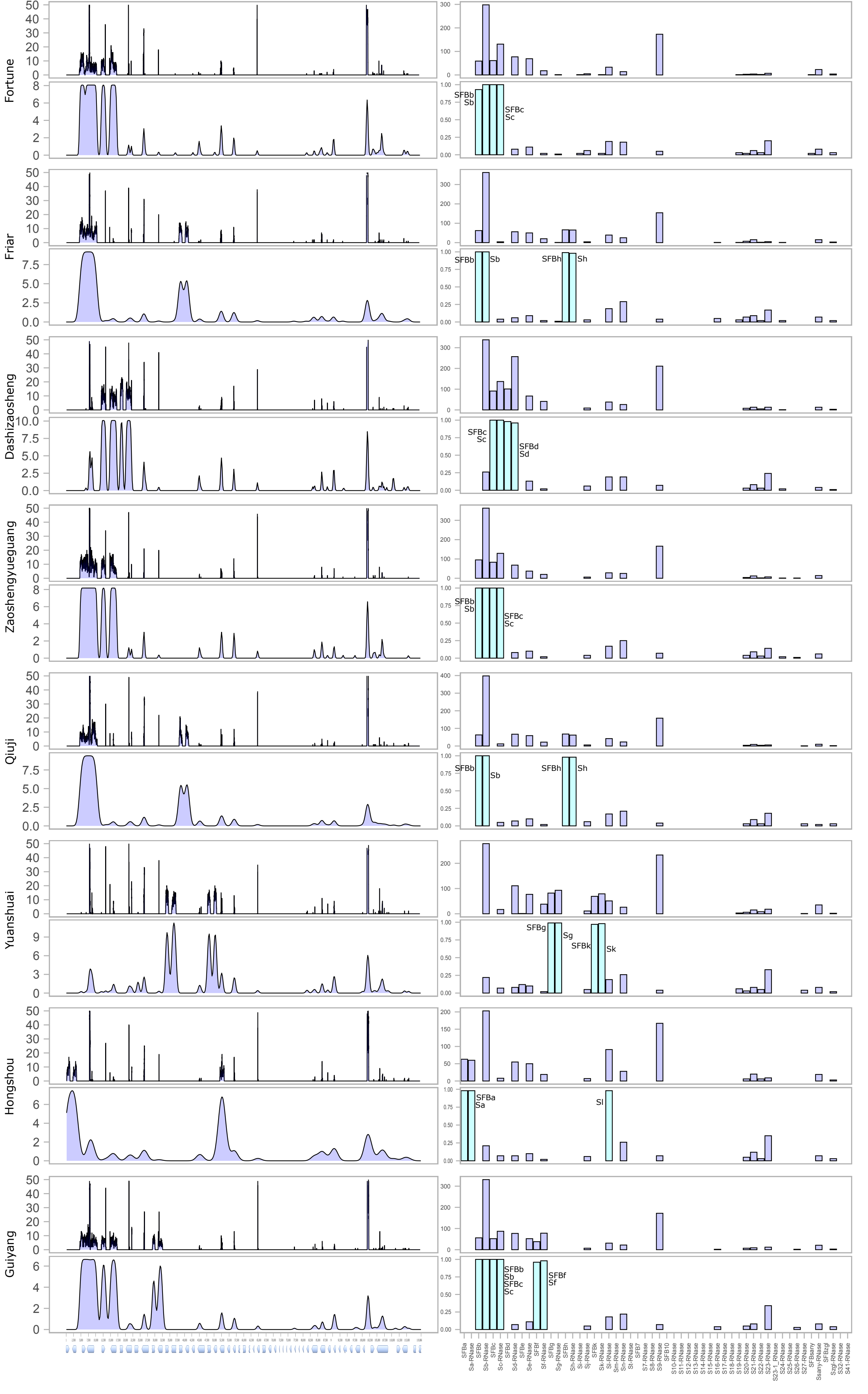

Fortune
SFBb
Sb
SFBc
Sc
Friar
SFBb
Sb
SFBh
Sh
Dashizaosheng
SFBc
Sc
SFBd
Sd
Zaoshengyueguang
SFBb
Sb
SFBc
Sc
Qiuji
SFBb
Sb
SFBh
Sh
Yuanshuai
SFBg
Sg
SFBk
Sk
Hongshou
SFBa
Sa
Sl
Guiyang
SFBb
Sb
SFBc
Sc
SFBf
Sf
SFBa
Sa-RNase
SFBb
Sb-RNase
SFBc
Sc-RNase
SFBd
Sd-RNase
SFBe
Se-RNase
SFBf
Sf-RNase
SFBg
Sg-RNase
SFBh
Sh-RNase
SFBi
Si-RNase
Sj-RNase
SFBk
Sk-RNase
Sl-RNase
Sm-RNase
Sn-RNase
St-RNase
SFB7
S7-RNase
S8-RNase
S9-RNase
SFB10
S10-RNase
S11-RNase
S12-RNase
S13-RNase
S14-RNase
S15-RNase
S16-RNase
S17-RNase
S18-RNase
S19-RNase
S20-RNase
S21-RNase
S22-RNase
S23-RNase
S23-1_RNase
S24-RNase
S25-RNase
S26-RNase
S27-RNase
SFBany
Ssany-RNase
SFBzgl
Szgl-RNase
S32-RNase
S41-RNase

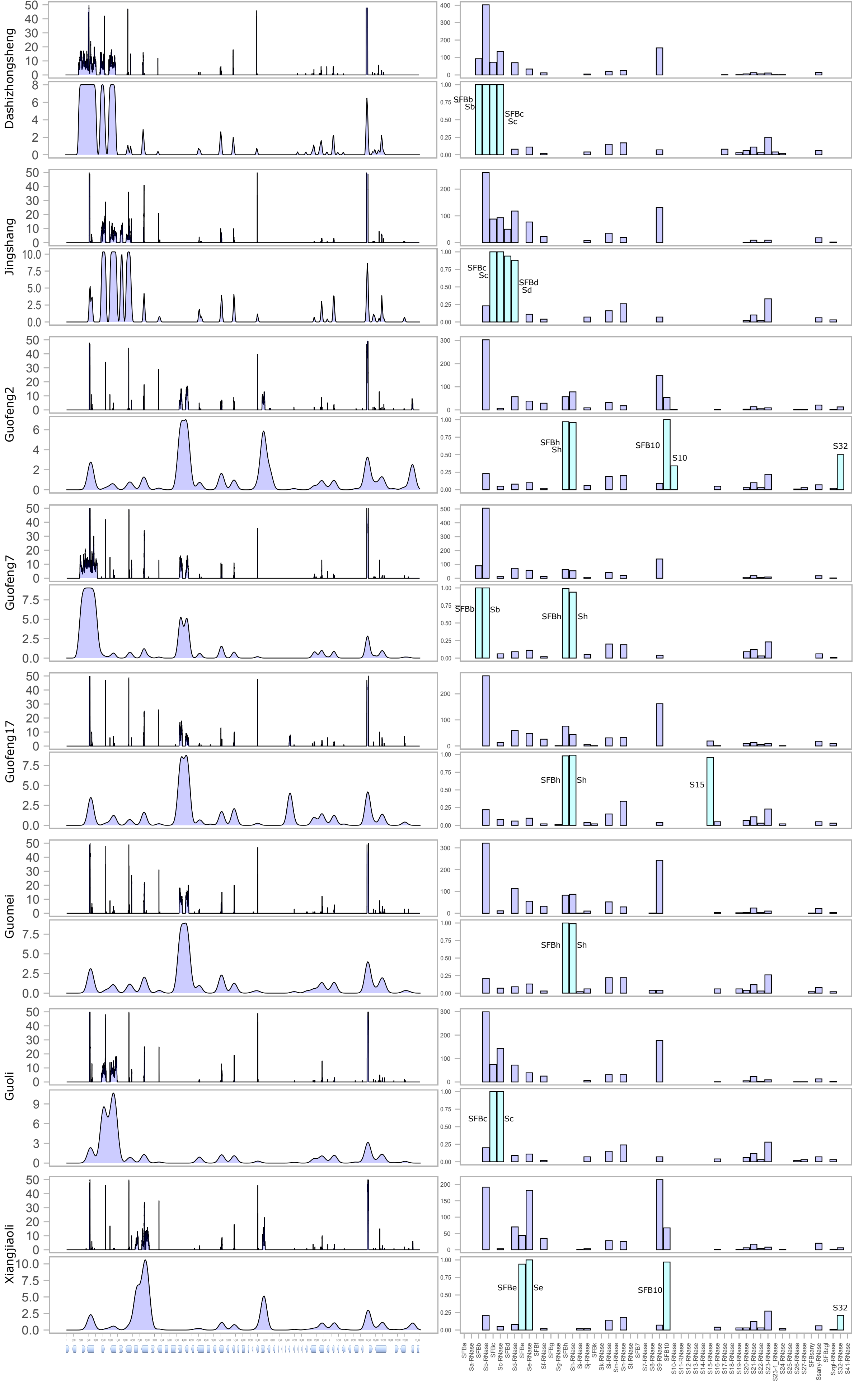
Dashizhongsheng
Jingshang
Guofeng2
Guofeng7
Guofeng17
Guomei
Guoli
Xiangjiaoli
SFBb
Sb
SFBc
Sc
SFBd
Sd
SFBh
Sh
SFB10
S10
S32
S15
SFBe
Se
SFBa
Sa-RNase
SFBb
Sb-RNase
SFBc
Sc-RNase
SFBd
Sd-RNase
SFBe
Se-RNase
SFBf
Sf-RNase
SFBg
Sg-RNase
SFBh
Sh-RNase
Si-RNase
Sj-RNase
SFBk
Sk-RNase
Sl-RNase
Sm-RNase
Sn-RNase
St-RNase
SFB7
S7-RNase
S8-RNase
S9-RNase
SFB10
S10-RNase
S11-RNase
S12-RNase
S13-RNase
S14-RNase
S15-RNase
S16-RNase
S17-RNase
S18-RNase
S19-RNase
S20-RNase
S21-RNase
S22-RNase
S23-RNase
S23-1_RNase
S24-RNase
S25-RNase
S26-RNase
S27-RNase
SFBsany
Ssany-RNase
SFBzgl
Szgl-RNase
S32-RNase
S41-RNase

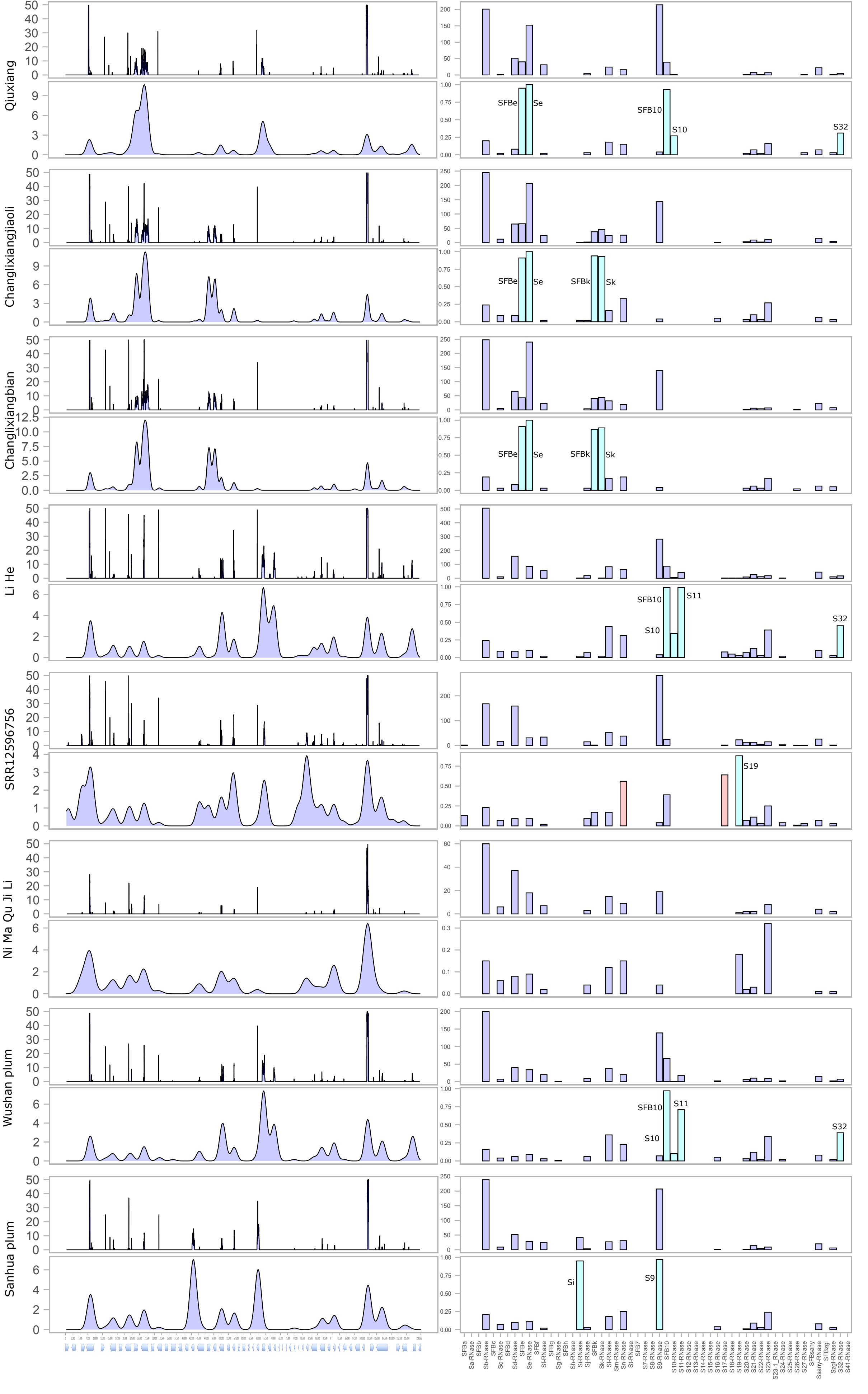

Qiuxiang
SFBe
Se
SFB10
S10
S32
Changlixiangjiaoli
SFBk
Sk
Changlixiangbian
Li He
S11
SRR12596756
S19
Ni Ma Qu Ji Li
Wushan plum
Sanhua plum
Si
S9
SFBa
Sa-RNase
SFBb
Sb-RNase
SFBc
Sc-RNase
SFBd
Sd-RNase
SFBe
Se-RNase
SFBf
Sf-RNase
SFBg
Sg-RNase
SFBh
Sh-RNase
Si-RNase
Sj-RNase
SFBk
Sk-RNase
Sl-RNase
Sm-RNase
Sn-RNase
St-RNase
SFB7
S7-RNase
S8-RNase
S9-RNase
SFB10
S10-RNase
S11-RNase
S12-RNase
S13-RNase
S14-RNase
S15-RNase
S16-RNase
S17-RNase
S18-RNase
S19-RNase
S20-RNase
S21-RNase
S22-RNase
S23-RNase
S23-1_RNase
S24-RNase
S25-RNase
S26-RNase
S27-RNase
SFBsany
Ssany-RNase
SFBzgl
Szgl-RNase
S32-RNase
S41-RNase

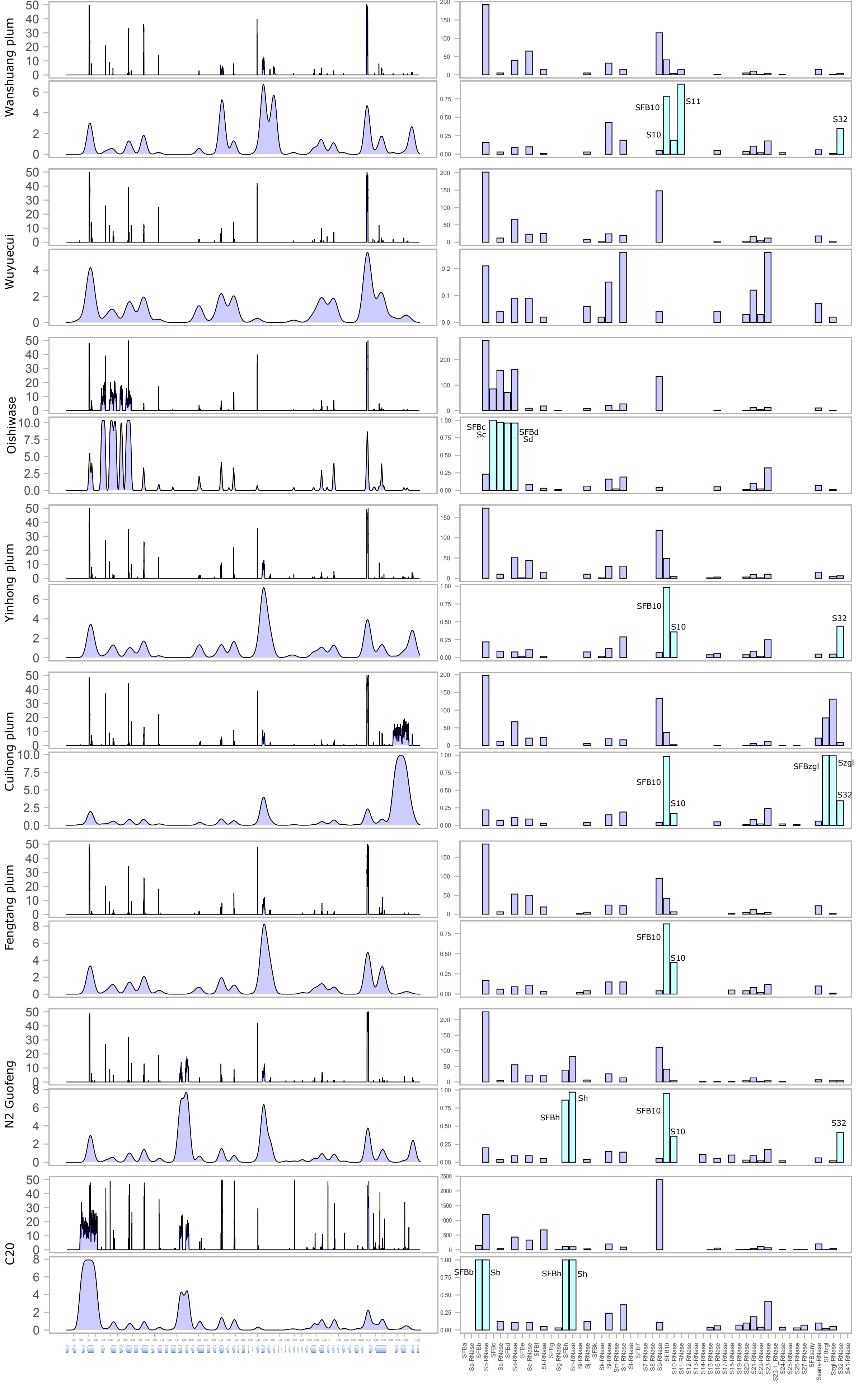
Wanshuang plum
SFB10
S11
S10
S32
Wuyuecui
Oishiwase
SFBc
Sc
SFBd
Sd
Yinhong plum
SFB10
S10
S32
Cuihong plum
SFB10
S10
SFBzgl
Szgl
S32
Fengtang plum
SFB10
S10
N2 Guofeng
SFBh
Sh
SFB10
S10
S32
C20
SFBb
Sb
SFBh
Sh
SFBa
Sa-RNase
SFBb
Sb-RNase
SFBc
Sc-RNase
SFBd
Sd-RNase
SFBe
Se-RNase
SFBf
Sf-RNase
SFBg
Sg-RNase
SFBh
Sh-RNase
Si-RNase
Sj-RNase
SFBk
Sk-RNase
Sl-RNase
Sm-RNase
Sn-RNase
St-RNase
SFB7
S7-RNase
S8-RNase
S9-RNase
SFB10
S10-RNase
S11-RNase
S12-RNase
S13-RNase
S14-RNase
S15-RNase
S16-RNase
S17-RNase
S18-RNase
S19-RNase
S20-RNase
S21-RNase
S22-RNase
S23-RNase
S23-1_RNase
S24-RNase
S25-RNase
S26-RNase
S27-RNase
SFBsany
Ssany-RNase
SFBzgl
Szgl-RNase
S32-RNase
S41-RNase

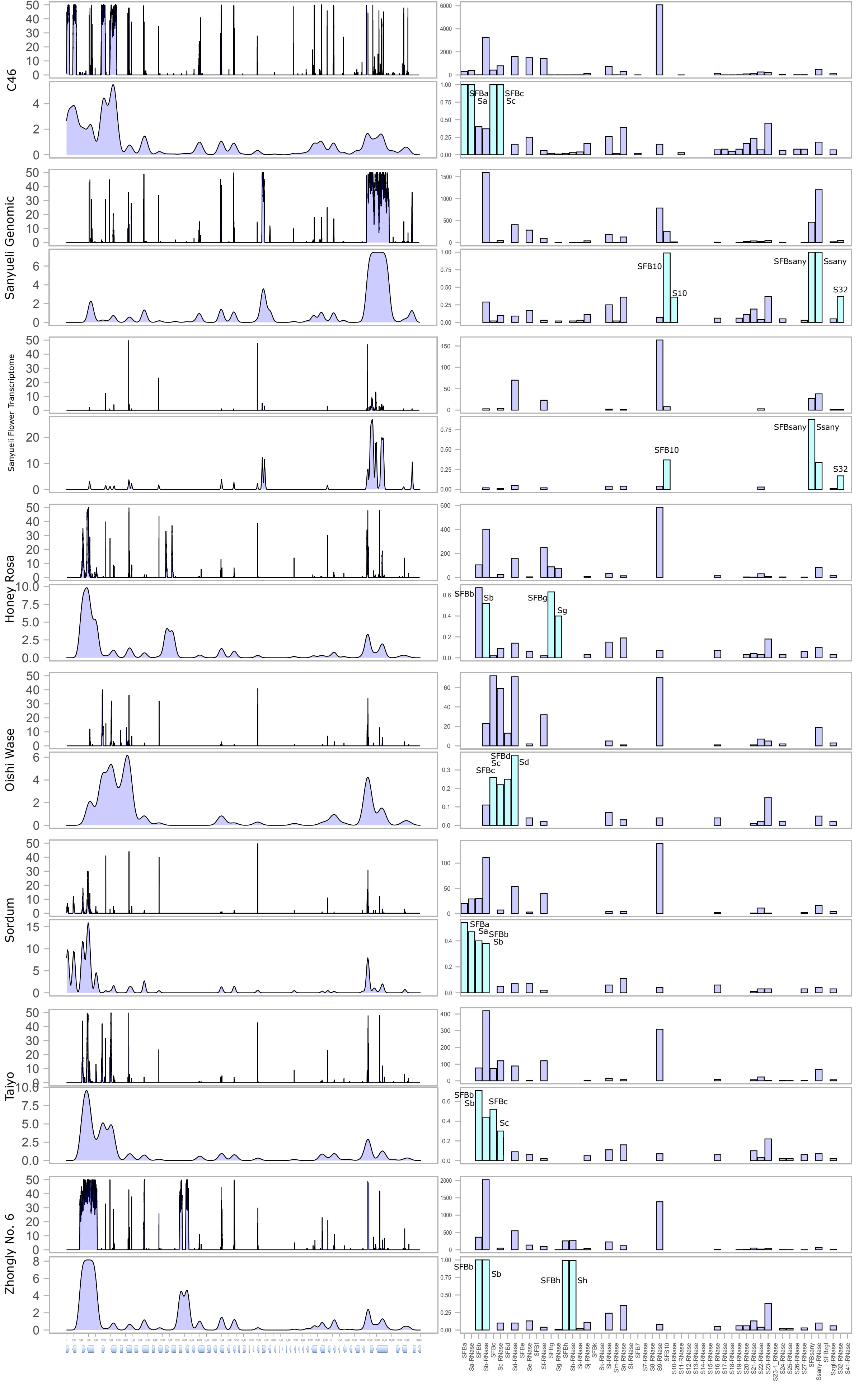
C46
Sanyueli Genomic
Sanyueli Flower Transcriptome
Honey Rosa
Oishi Wase
Sordum
Taiyo
Zhongly No. 6
SFBa
Sa
SFBc
Sc
SFB10
S10
SFBsany
Ssany
S32
SFBb
Sb
SFBg
Sg
SFBd
SFBh
Sh
